# Hypothalamic Neurogenesis as an Adaptive Metabolic Mechanism

**DOI:** 10.3389/fnins.2017.00190

**Published:** 2017-04-05

**Authors:** Antonia Recabal, Teresa Caprile, María de los Angeles García-Robles

**Affiliations:** ^1^Laboratorio de Biología Celular, Departamento de Biología Celular, Facultad de Ciencias Biológicas, Universidad de ConcepciónConcepción, Chile; ^2^Laboratorio de Guía Axonal, Departamento de Biología Celular, Facultad de Ciencias Biológicas, Universidad de ConcepciónConcepción, Chile

**Keywords:** tanycytes, hypothalamus, glucosensing, feeding behavior, neurogenesis

## Abstract

In the adult brain, well-characterized neurogenic niches are located in the subventricular zone (SVZ) of the lateral ventricles and in the subgranular zone (SGZ) of the hippocampus. In both regions, neural precursor cells (NPCs) share markers of embryonic radial glia and astroglial cells, and *in vitro* clonal expansion of these cells leads to neurosphere formation. It has also been more recently demonstrated that neurogenesis occurs in the adult hypothalamus, a brain structure that integrates peripheral signals to control energy balance and dietary intake. The NPCs of this region, termed tanycytes, are ependymal-glial cells, which comprise the walls of the infundibular recess of the third ventricle and contact the median eminence. Thus, tanycytes are in a privileged position to detect hormonal, nutritional and mitogenic signals. Recent studies reveal that in response to nutritional signals, tanycytes are capable of differentiating into orexigenic or anorexigenic neurons, suggesting that these cells are crucial for control of feeding behavior. In this review, we discuss evidence, which suggests that hypothalamic neurogenesis may act as an additional adaptive mechanism in order to respond to changes in diet.

## Introduction

Postnatal neurogenesis corresponds to the series of events that lead to the production of new neurons in the adult brain, from precursor cell division to the survival and functional integration of newly differentiated neurons (Lledo et al., 2006). In the adult brain under normal conditions, well-characterized niches are restricted to the subventricular zone (SVZ) of lateral ventricles (Doetsch et al., 1999) and the hippocampal subgranular zone (SZG; Altman and Das, 1965). Experiments also suggest the existence of constitutive neurogenesis in close proximity to circumventricular organs (Hourai and Miyata, 2013).

Neurogenic cells present in the SVZ and SGZ share the following features: (i) astroglial marker expression, including glial fibrillary acidic protein (GFAP) and GLutamate ASpartate Transporter (GLAST) (Platel et al., 2009); (ii) stem cell marker expression, such as nestin and SOX2 (Imayoshi et al., 2008); and (iii) *in vitro* clonal expansion resulting in neurosphere formation (Lledo et al., 2006; Kriegstein and Alvarez-Buylla, 2009).

Neurogenesis also occurs in the adult hypothalamus (Evans et al., 2002; Cheng, 2013), a brain structure located at the base of the diencephalon, close to the third ventricle (3V) and in close contact to the median eminence (ME), a circumventricular organ. The present review will discuss the cell origin of the hypothalamic newborn neurons and its physiological role in the energy balance.

## Hypothalamus and energy balance

The hypothalamus is the main regulator of energy balance and instinctive behaviors, including food intake. In the hypothalamus, the arcuate nucleus (AN) is composed of clustered neuronal populations that inhibit or initiate food intake through the release of anorexigenic or orexigenic peptides, respectively (Schwartz et al., 2000). There is great interest in understanding the precise molecular and cellular mechanisms that control glucosensing, especially given that diabetes and obesity may be induced by a dysregulation in this process (Elizondo-Vega et al., 2016).

**Figure 1:**
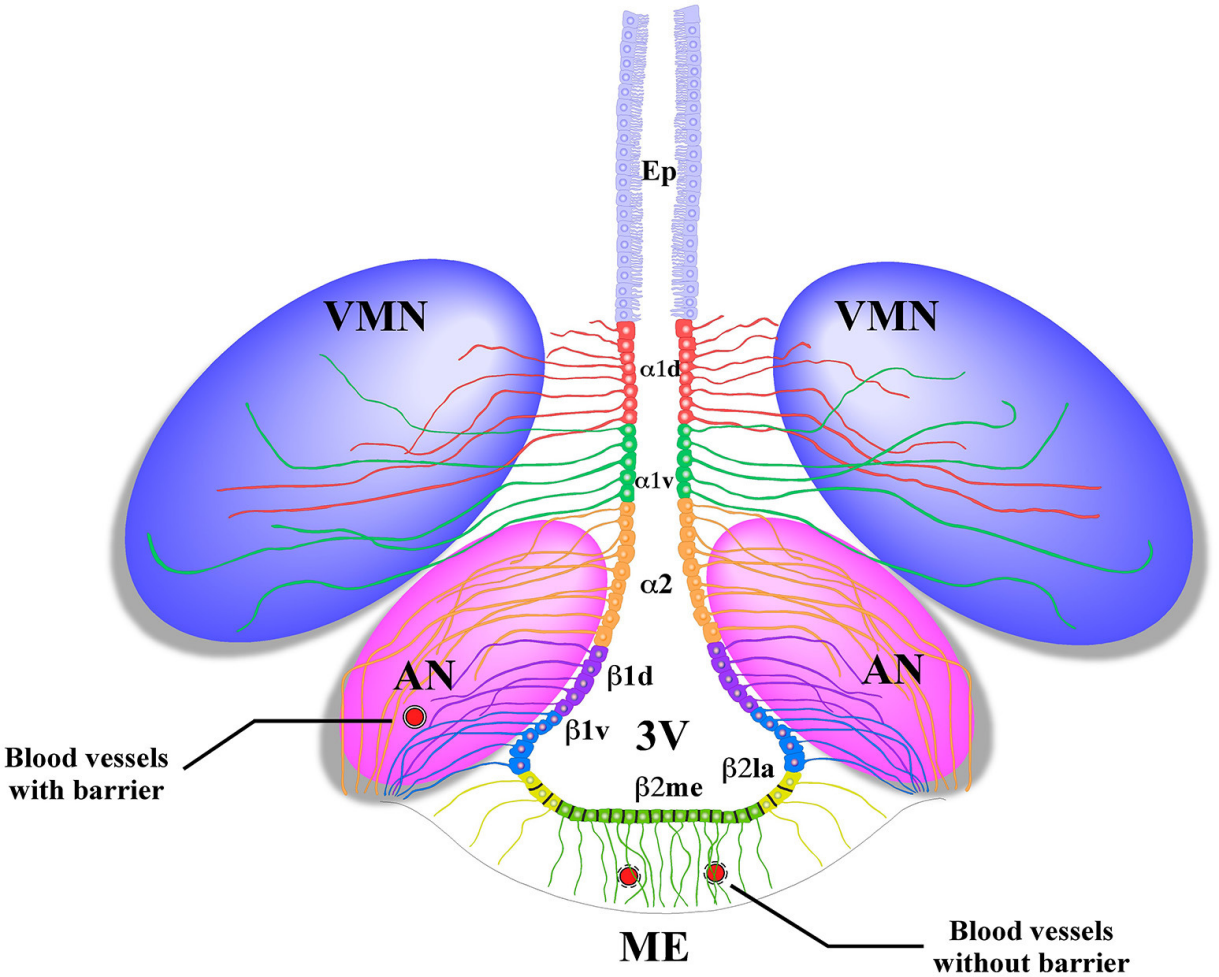
**Schematic representation of the basal hypothalamus**. Ciliated ependymocytes (Ep) line the dorsal wall of the 3V. The α1d-tanycytes (α1d) and α1v-tanycytes (α1v) have long projections that make contact with the neurons of the VMN. The α2-tancycytes (α2) β1d-tanycytes (β1d) and β1v-tanycytes (β1v) make projections to the AN, making contact with orexigenic and anorexigenic neurons and blood vessels. In the floor of the 3V, the β2 lateral-tanycytes (β2la) and β2 medial-tanycytes (β2me) are joined by tight junctions forming part of the median eminence (ME)-cerebrospinal fluid (CSF) barrier, and their projections make contact with the fenestrated blood vessels of the ME. Redrawn from a previously published (Elizondo-Vega et al., 2015).

Radial glial-like tanycytes surround the lateral walls of the infundibular recess. Their apical poles contact the cerebrospinal fluid (CSF), and the basal extensions project into the AN (Flament-Durand and Brion, 1985). Tanycytes are classified into four main groups on the basis of differences in their localization and gene expression: α1, α2 (Robins et al., 2013), β1, and β2 (Rodríguez et al., 2005). α2 and β1 tanycytes are located in the lateral walls of the 3V and contact anorexigenic and orexigenic neurons through their extensive processes. β2-tanycytes cover the floor of the 3V and present tight junctions that form the CSF-ME barrier and extend their projections inside the ME (Figure 1); their tight junctions and cellular contacts can change, depending on the metabolic state of the organism (Langlet et al., 2013).

It has been postulated that tanycytes function as neuromodulating cells, since they regulate the availability of hormones, such as leptin (Balland et al., 2014) and ghrelin (Collden et al., 2015), from peripheral tissues to neurons of the AN. They also express the molecular machinery that permits glucose sensing (García et al., 2003; Cortés-Campos et al., 2011; Orellana et al., 2012) and transmit signals to AN neurons (Elizondo-Vega et al., 2016). Due to their privileged position in the hypothalamus, tanycytes can also detect mitogenic and neurodifferentiating signals present in the peripheral blood or in the CSF (Robins et al., 2013; Chaker et al., 2016).

## Tanycytes as hypothalamic neural precursor cells (NPCs)

Tanycytes have been suggested as possible hypothalamic precursors since they share the characteristics of the neuronal precursors of the SVZ and SGZ. Specifically, they express GFAP (Haan et al., 2013), GLAST (Robins et al., 2013), nestin and SOX2 (Lee et al., 2012; Li et al., 2012). Tanycytes also express the multipotent cell markers, UGS148 (Ma et al., 2015) and FGF10 (Haan et al., 2013), and are capable of forming neurospheres (Robins et al., 2013). Different populations of tanycytes have different neurosphere-forming capabilities; dorsal α2 tanycytes actively proliferate and form new neurospheres after cell isolation, while β tanycytes do not form neurospheres (Robins et al., 2013).

In lineage-tracing experiments using a Cre/lox system in which recombinase were expressed in tanycytes under the control of promoters, such as nestin (Lee and Blackshaw, 2012), glast (Robins et al., 2013), fgf10 (Haan et al., 2013), and prss56 (Jourdon et al., 2015), their role as neural stem cells was confirmed. In these transgenic mice, the constitutive expression of a reporter gene facilitates tanycyte tracking over time. The use of transgenic Cre/lox together with the intraperitoneal (ip) or intracerebroventricular (icv) delivery of BrdU has shown the self-renewal of tanycytes and their differentiation into other cell types, including mature neurons that respond to peripheral signals (Table 1).

**Table 1:** **Summary of the animal models features, dietary factors involved in hypothalamic neurogenesis and its implication in the energy balance**.

**Mice sex**	**Transgenic**	**Age**	**BrdU administration**	**Analisis time**	**Newborn cells origin**	**Neurogenesis rate under normal conditions**	**Newborn neurons phenotype**	**Functional activity of newborn neurons**	**Treatment**	**Effect on the energy balance**	**Reference**
Male	None or B6.V-Lep^ob^/J (ob/ob)	P56	icv infusion for 7 days	~P116	Widespread	20.7% of BrdU+ cells express Hu	NPY+ POMC+	pSTAT3 in response to leptin	HFD for 2 months + CNTF icv for 7 days	↑ proliferation ↓ body weight	Kokoeva et al., 2005
									HFD for 2 months + AraC icv for 7 days	↓ proliferation ↑ body weight as the control	
Male	AgRP^Cre/+^:Tfam^flox/flox^ AgRP^Cre/+^:R26^lacZ/+^	P84	icv infusion for 6 weeks	~P126	AN	7.2–11.8% of Ki-67+ or PCNA+ cells express LacZ in the mutants	AgRP+ POMC+ in mutants	pSTAT3 in response to leptin	AraC inhibited proliferation in mutants	↓ proliferation ↓ food intake ↓ body adiposity	Pierce and Xu, 2010
Female	Nestin^CreER^:R26YFP	P45	ip for 9 days	P75	ME (β-tanycytes)	7.8% of YFP+ cells express Hu	NPY+ POMC+	pSTAT3 in response to leptin c-fos expression in response to fasting	HFD for 30 days Guided irradiation	↑ neurogenesis in the ME ↓ proliferation ↓ weight gain on HFD	Lee and Blackshaw, 2012
Male	None	P56	icv infusion for 7 days or less	~P60	AN	93.4% of BrdU+ cells express NeuN after 2 weeks	POMC+		HFD for 3 weeks	↑ POMC generated neurons ↓ weight gain ↓ proliferation ↑ body weight ↑ adiposity	Gouazé et al., 2013
									HFD for 3 weeks + AraC icv for 3 weeks		
Both	Fgf10^nlacZ/+^ Fgf10^CreERT2^: R26^lacZ^ Fgf10^CreERT2^:R26^Tom^	P28-P60	via drinking water	P31-103	Tanycytes	78/89 of Xgal+ cells express NeuN	NPY+	pSTAT3 in response to leptin c-fos expression in response to fasting	ip and diet tamoxifen for 3–8 days	N.S.	Haan et al., 2013
N.S.	Glast^CreERT2^: R26^lacZ^ Glast^CreERT2^: R26^GFP^	P42-P56	icv infusion for 7 days	6 weeks 9 months	α-tanycytes	1–2% of reporter+ cells express NeuN	N.S.	N.S.	ip tamoxifen for 10 days	N.S.	Robins et al., 2013
Both	None	P42	ip for 8 days	P75	ME (β-tanycytes) AN	1.2–1.5% of BrdU+ cells express Hu	N.S.	N.S.	HFD for 4.5 months	**Female** ↑ neurogenesis in the ME ↓ neurogenesis in the AN **Male** ↓ neurogenesis in the AN	Lee et al., 2014
Female	None	P70-P84	icv infusion for 7 days	~P104-118	Widespread	N.S.	ERα+	pSTAT3 in response to leptin	HFD for 34 days HFD + estradiol for 34 days	↑ proliferation in the AN/VMH ↓ proliferation in the AN/VMH	Bless et al., 2016
Male	Nestin^CreERT2^: CAG-R26^tdTomato/+^: IGF-1R^flox/flox^	P90	N.S.	~P120-P480	α-tanycytes	273 of Tomato+ cells express NeuN	GHRH+	Glutamate and GABA receptors expressing neurons	Genetic deletion of IGF-1R	↑ neurogenesis in all hypothalamic nuclei	Chaker et al., 2016

*N.S., not specified; pSTAT3, phosphorilated STAT3*.

After successive divisions, hypothalamic progenitors begin to express proteins characteristic of migrating, immature neurons, including neural cell adhesion molecule with polysialic acid modification (PSA-NCAM) (Bonfanti et al., 1992); the microtubule binding protein, doublecortin (DCX), has also been found in human hypothalamic slices (Batailler et al., 2014). Some of the neuroblasts that originated from tanycytes are able to migrate to the AN, differentiate to an orexigenic or anorexigenic neurons, and respond to peripheral signals, such as leptin (Kokoeva et al., 2005; Lee and Blackshaw, 2012). This suggests that the rate of neuronal renewal is not merely restorative; it consists of an adaptive mechanism in response to metabolic changes imposed by the environment and/or the internal state of the organism (Lledo et al., 2006).

## Hypothalamic neurogenesis as an adaptive metabolic mechanism

The normal proportion of newborn neurons among newborn cells in the adult rodent hypothalamus is lower (1–37%; Migaud et al., 2010) than in the SGZ and SVZ (70–100%; (Lledo et al., 2006)), but its rate increase after icv administration of ciliary neurotrophic factor (CNTF) (Kokoeva et al., 2005), insulin-like growth factor (IGF) (Pérez-Martín et al., 2010), brain derived neurotrophic factor (BNDF) (Pencea et al., 2001), and fibroblast growth factor 2 (FGF2) (Xu et al., 2005; Robins et al., 2013). These factors enhance proliferation of cells that are in proximity of the 3V, both into the parenchyma or ventricular zone. Specifically, it has been shown that FGF2 (Robins et al., 2013) and IGF-1R (Chaker et al., 2016) are directly involved in the regulation of α-tanycyte proliferation, whereas circulating lipids promote β-tanycyte proliferation in female rats. The type of tanycyte that act as NPC may seem controversial, but the source of the triggering elements and the sex of the model studied must be considered (Lee et al., 2014).

Changes in metabolic conditions can also modify the proliferation of hypothalamic neuronal precursors (Table 1), including high temperatures (Matsuzaki et al., 2009), physical activity (Niwa et al., 2016), and a high fat diet (HFD) (Kokoeva et al., 2005; Lee and Blackshaw, 2012; Gouazé et al., 2013; Nascimento et al., 2016). During prenatal neurogenesis, *in utero* exposure to a HFD stimulates the production of orexigenic hypothalamic neurons (Chang et al., 2008), which causes changes in behavior and physiological conditions that extend into adulthood as demonstrated by the increased body weight and caloric intake observed in P70 (Chang et al., 2008).

Other experiments using adult rodents under a HFD have shown the following.

- Anorexigenic neurogenesis is accelerated, preventing the weight gain and fat mass induced by the change in diet (Gouazé et al., 2013). Dietary or direct 3V injection of polyunsaturated fatty acids, such as docosahexaenoic acid (DHA), increases the generation of pro-opiomelanocortin (POMC)-expressing neurons, possibly through the interaction of GPR40 fatty acids receptors (Nascimento et al., 2016). Female but not male mice consuming a HFD had increased cell proliferation that was attenuated by estradiol in the AN (Bless et al., 2016).
- Chronic exposure to a HFD leads to the loss of mature hypothalamic orexigenic and anorexigenic neurons (Moraes et al., 2009), as well as loss of hypothalamic neuronal precursors (Li et al., 2012). The inflammatory microenvironment activated in preobesity and prediabetes impairs the survival of hypothalamic neuronal progenitors upon activation of IκB kinase B/nuclear κB factor (Li et al., 2012).
- Simultaneous icv infusion of cytosine-β-D-arabinofuranoside (AraC) is sufficient to cause an exaggerated increase in body weight (Kokoeva et al., 2005; Gouazé et al., 2013), implying that hypothalamic neurogenesis may restore energy balance. In contrast, inhibition of mitosis in the ventromedial hypothalamus of female mice increases energy expenditure and diminishes body weight, suggesting that neuronal differentiation due to a HFD promotes energy storage (Lee and Blackshaw, 2012; Lee et al., 2014).
- Transgenic mice that have a progressive degeneration of AgRP orexigenic neurons due the genetic deletion of the *mitochondrial transcription factor A* (*Tfam*) originate a new subset of AgRP neurons, a fact that explains why mutant mice do not exhibit decreased body weight in response to neurodegeneration (Pierce and Xu, 2010).

It should be noted that experiments differ in the promoter used, the sex and age of the animals and the chase term (Table 1). The added neurons may play a different role in the regulation of dietary intake and body weight depending on the hypothalamic region they established and the factors mentioned (Lee et al., 2014).

## Conclusion

Hypothalamic neurogenesis can be stimulated by intrinsic factors, such as CSF-derived mitogenic molecules or peripheral factors that cross the ME. Additionally, this event can be promoted by environmental changes in the diet (e.g., HFD and calorie restriction) or neurodegeneration (e.g., genetically induced death of AgRP neurons). Both conditions promote the generation of new neurons as part of a response to restore energy balance prior to the development of metabolic diseases that prevent the generation and proper development of hypothalamic NSCs. The specific mechanisms that link changes in the diet with the proliferation of tanycytes remain unknown, but they may involve their well-known nutrient chemosensitive machinery and/or their metabolic coupling with AN neurons that control appetite.

## Author contributions

AR conceived the review focus, conducted the literature review, and summarized and finalized the manuscript. TC and MG reviewed the literature, wrote the first draft, and finalized the manuscript. All authors approved final version of manuscript.

## Funding

This study was funded by the National Fund for Scientific and Technological Development (FONDECYT number 1140677).

### Conflict of interest statement

The authors declare that the research was conducted in the absence of any commercial or financial relationships that could be construed as a potential conflict of interest.
